# Finding Oxygen Reservoir by Using Extremely Small Test Cell Structure for Resistive Random Access Memory with Replaceable Bottom Electrode

**DOI:** 10.1038/srep18442

**Published:** 2015-12-22

**Authors:** Kentaro Kinoshita, Sang-Gyu Koh, Takumi Moriyama, Satoru Kishida

**Affiliations:** 1 Department of Information and Electronics, Graduate School of Engineering, Tottori University, 4-101 Koyama-Minami, Tottori 680-8552, Japan; 2 Tottori Integrated Frontier Research Center, 4-101 Koyama-Minami, Tottori 680-8552, Japan; 3 Tottori Univ. Electronic Display Research Center, 4-101 Koyama-Minami, Tottori 680-8552, Japan

## Abstract

Although the presence of an oxygen reservoir (OR) is assumed in many models that explain resistive switching of resistive random access memory (ReRAM) with electrode/metal oxide (MO)/electrode structures, the location of OR is not clear. We have previously reported a method, which involved the use of an AFM cantilever, for preparing an extremely small ReRAM cell that has a removable bottom electrode (BE). In this study, we used this cell structure to specify the location of OR. Because an anode is often assumed to work as OR, we investigated the effect of changing anodes without changing the MO layer and the cathode on the occurrence of reset. It was found that the reset occurred independently of the catalytic ability and Gibbs free energy (ΔG) of the anode. Our proposed structure enabled to determine that the reset was caused by repairing oxygen vacancies of which a filament consists due to the migration of oxygen ions from the surrounding area when high ΔG anode metal is used, whereas by oxidizing the anode due to the migration of oxygen ions from the MO layer when low ΔG anode metal is used, suggesting the location of OR depends on ΔG of the anode.

Models in which resistive switching of an electrode/metal oxide (MO)/electrode structure is caused by the thermally activated migration of oxygen ions are being widely accepted. In these models, a conductive filament (CF) that is formed in the MO layer consists of oxygen vacancies, and resistive switching is caused by the generation and repair of oxygen vacancies (V_O_’s)^1^,^2^,^3^,^4^,^5^,^6^,^7^,^8^,^9^. This means that resistive switching is caused by exchanging oxygen ions between the (CF) and the oxygen reservoir (OR). However, information about the OR is still incomplete. We do not even know the location of the OR; for example, is it located in the electrode or in the MO layer? The location of the OR should be elucidated to design the cell structure, including electrodes and MO materials for optimizing memory performance. Because many papers claim that an anode works as an OR^10^,^11^,^12^,^13^, it is advantageous for identifying the location of an OR if the anode can be replaced without changing the MO layer and the cathode.

Recently, we reported a method, which involved the use of an AFM cantilever, for preparing an extremely small ReRAM cell that has a removable bottom electrode (BE)^14^. First, a MO layer is deposited on the surface of a Pt-coated cantilever that works as a top electrode (TE). Next, by contacting a BE with the cantilever, a tiny ReRAM cell structure is formed in the contact area. This method enables anode replacement at any time by simply moving the cantilever from one BE to another BE.

In this study, the influence of changing the anode material without changing the MO layer and the cathode of the cathode/MO/anode structure on the presence and absence of a reset was investigated by utilizing our proposed cell structure. As a result, the MO layer is suggested to be the OR when a metal with a high Gibbs energy of formation reaction is used as the anode, whereas to be the anode itself when a metal with a low Gibbs energy is used as the anode.

## Results and Discussion

**Figure 1:**
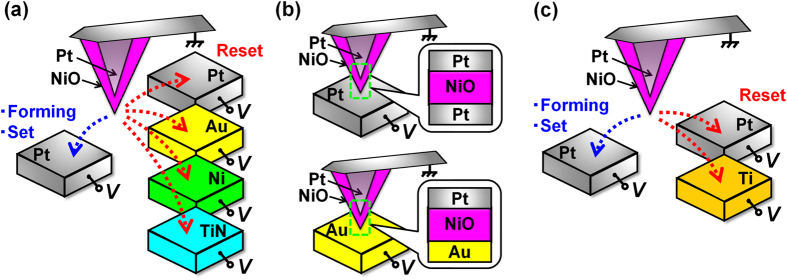
(**a**) A Pt-, Au-, Ni-, and TiN-BE, which were used only for reset, and another common Pt-BE, which was used only for forming and set, were formed on the same substrate. (**b**) Schematics explaining that the ReRAM cell is formed at the contact area between the cantilever and the BE. (**c**) A Ti- and Pt-BE, which were used only for reset, and another common Pt-BE, which was used only for forming and set, were formed on the same substrate.

Cantilevers on which a Pt/NiO and an Au/NiO structure were formed were prepared and will be described respectively as a Pt cantilever and an Au cantilever hereafter. On the other hand, Pt-, Au-, Ni-, and TiN-BE(R)s with a thickness of 100 nm were prepared on the same SiO_2_/Si substrate, as shown in Fig. 1(a). A Pt-TE/NiO/X-BE (X = Pt, Au, Ni, or TiN) structure was formed by contacting the X-BE with the cantilever and the area of the structure was estimated to be less than 10 nm in diameter^14^. For example, by contacting the Pt-BE with the Pt cantilever, a Pt-TE/NiO/Pt-BE structure is formed in the contact area, as shown in the upper schematic in Fig. 1(b). On the other hand, by contacting the Au-BE with the Pt cantilever, a Pt-TE/NiO/Au-BE structure is formed in the contact area, as shown in the lower schematic in Fig. 1(b). These four BEs were used for reset, and will be denoted hereafter as X-BE(R) (X = Pt, Au, Ni, or TiN). On the other hand, another common Pt-BE was also prepared on the same substrate for forming and set, and will be denoted hereafter as Pt-BE(FS). In addition, another substrate on which a Ti-BE for reset (Ti-BE(R)), Pt-BE(R), and Pt-BE(FS) was formed was also prepared, as shown in Fig. 1(c).

**Figure 2:**
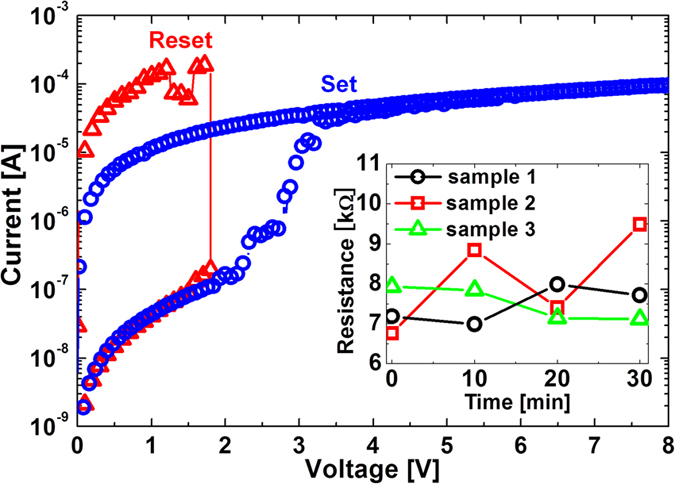
*I*–*V* characteristics of a Pt-TE/NiO/Pt-BE structure that was measured by voltage sweep. Time dependences of resistances in the low resistance state for three different samples are shown in the inset.

Figure 2 shows the current-voltage (*I-V*) characteristics of a Pt-TE/NiO/Pt-BE structure that was measured by sweeping the bias voltage. Unipolar switching was confirmed, where set and reset occurred on Pt-BE(FS) and Pt-BE(R), respectively. We investigated the dependence of BE materials on the occurrence of reset by applying pulse voltages to reduce the damage due to excess Joule heating. In addition, time dependences of resistances in the low resistance state for three different samples are shown in the inset of Fig. 2. Circles, squares, and triangles show time dependences of resistances for three different samples which were prepared under the same conditions. Although the low resistance state is retained at least more than 30 min, the resistance randomly changes with the drift of cantilever, suggesting that the measurement conditions continuously change in a strict sense. Therefore, we avoid discussing the electrode material dependence of the resistance and the switching voltage, and restrict ourselves to discussion about the occurrence of resistive switching. The results of the investigation are presented later in the paper.

**Figure 3:**
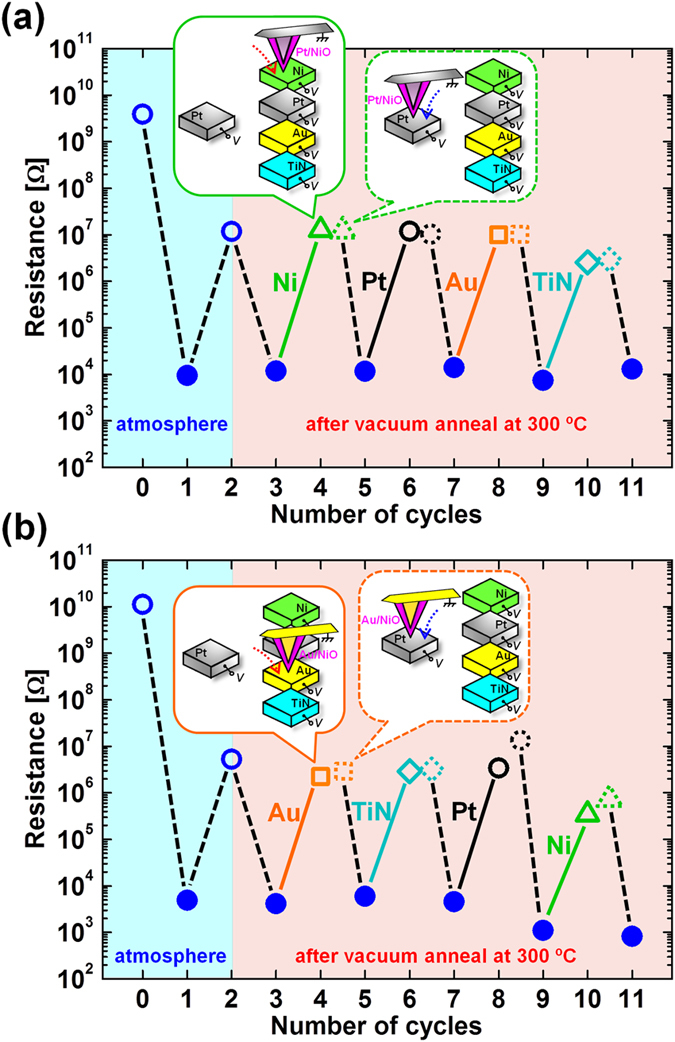
(**a**) Sequential resistive switching using the Pt cantilever: forming on Pt-BE(FS) ⇒ reset on Pt-BE(R) ⇒ vacuum anneal for 10 min at 300 °C ⇒ set on Pt-BE(FS) ⇒ reset on Ni-BE(R) ⇒ set on Pt-BE(FS) ⇒ reset on Pt-BE(R) ⇒ set on Pt-BE(FS) ⇒ reset on Au-BE(R) ⇒ set on Pt-BE(FS) ⇒ reset on TiN -BE(R), where all the measurements after the vacuum annealing were performed at RT without breaking the vacuum. Resistance values after set and reset are shown respectively by filled and open symbols. After the occurrence of reset on each BE(R) (Ni: open triangle, Pt: open circle, Au: open square, TiN: open diamond), resistance value was read out again on the Pt-BE(FS), and the readout value are shown by dotted-line symbols. (**b**) Sequential resistive switching using the Au cantilever: forming on Pt-BE(FS) ⇒ reset on Pt-BE(R) ⇒ vacuum anneal for 10 min at 300 °C ⇒ set on Pt-BE(FS) ⇒ reset on Au-BE(R) ⇒ set on Pt-BE(FS) ⇒ reset on TiN-BE(R) ⇒ set on Pt-BE(FS) ⇒ reset on Pt-BE(R) ⇒ set on Pt-BE(FS) ⇒ reset on Ni -BE(R), where all the measurements after the vacuum annealing were performed at RT without breaking the vacuum. Resistance values after set and reset are shown respectively by filled and open symbols. After the occurrence of reset on each BE(R) (Au: open square, TiN: open diamond, Pt: open circle, Ni: open triangle), resistance value was read out again on the Pt-BE(FS), and the readout value are shown by dotted-line symbols.

First, the cantilever was brought into contact with the Pt-BE(FS) in the atmosphere. After forming on the Pt-BE(FS), the cantilever was brought into contact with the Pt-BE(R), and the occurrence of the first reset was confirmed. Next, the chamber was evacuated to a pressure of 10^−4^ Pa, and all the BEs were annealed for 10 min at 300 °C in order to desorb water from the surface of the BEs to avoid field oxidation or reduction of the NiO layer and the BEs. After cooling the BEs to room temperature (RT), the cantilever was brought into contact with the Pt-BE(FS) again, and the occurrence of the first set was confirmed. Resistances before forming, after forming, after the first reset, and after the first set are denoted by the left four circles in Fig. 3(a), where resistance values read out after set (forming) and reset are shown respectively by filled and open symbols. Next, the cantilever was brought into contact with Ni-BE(R), and the occurrence of reset was confirmed. Next, the cantilever was brought into contact with Pt-BE(FS), and the occurrence of set was confirmed. In the same way, reset was attempted sequentially on each BE in the order of Ni-, Pt-, Au-, and TiN-BE(R) using the same cantilever at RT without breaking the vacuum. All the set processes were performed on the Pt-BE(FS) as described earlier. Reset was confirmed on all the BEs and, therefore, high resistance values were read out after the occurrence of reset as shown respectively by the open triangle, circle, square, and diamond in Fig. 3(a). Average pulse heights at which reset occurred were 1.43, 1.62, 1.48, and 1.13 V for Ni-, Pt-, Au-, and TiN-BE(R), respectively. After the occurrence of reset on each BE(R), the resistance value was read out again on the Pt-BE(FS), and the readout values are shown by the dotted-line symbols in Fig. 3(a). The high resistance values observed on the BE(R)s were retained after moving the cantilever to Pt-BE(FS).

The same measurement was performed by using a Au-cantilever instead of using a Pt-cantilever. The result is shown in Fig. 3(b). In this case, we also observed the occurrence of reset on all of the BE(R)s and the high resistance values observed on the BE(R)s were retained after moving the cantilever to Pt-BE(FS) as well as the case using the Pt-cantilever. Average pulse heights at which reset occurred were 1.15, 1.70, 1.20, and 1.33 V for Ni-, Pt-, Au-, and TiN-BE(R), respectively.

We discuss these results in terms of catalysis and Gibbs free energy of formation reaction (ΔG) of the BEs, where ΔG is defined as the difference between the Gibbs free energy of MO (PtO_2_, Au_2_O_3_, NiO, and TiO_2_) or metal nitride (TiN) and the Gibbs energy of pure metal (Pt, Au, Ni, and Ti). The presence of catalytic ability (in terms of the dissociation of oxygen) and ΔG at RT of the BEs are summarized in Table 1^15^. In addition to the presence of water on the BEs as described earlier, we have to consider some other absorbing species depending on BE materials. We focus on oxygen and the hydroxyl radical (OH) as possible absorbing species that enhance reset switching, because reset is generally thought to be caused by repairing a part of the V_O_’s that a CF comprises. Oxygen is known to be dissociatively-adsorbed on the surface of Pt, and the desorption temperatures of molecular and atomic oxygen from the Pt(111) surface are reported respectively to be 150 and 750 K^16^. On the other hand, OH is reported to be formed following the reaction formula^17^,^18^:


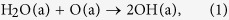


where H_2_O(a), O(a), and OH(a) mean H_2_O, O, and OH absorbed on the surface of Pt. However, the desorption temperature of H_2_O is reported to be approximately 210 K, and thus no OH is present on the surface of Pt-BE after vacuum annealing for 10 min at 300 °C ( = 573 K)^17^. Therefore, we have to consider the presence of atomic oxygen among oxidizing species. In addition, no catalytic effect on oxygen has been reported on the surfaces of Au and TiN. Therefore, our result suggests that reset occurs independently of the presence or absence of catalytic ability of anodes, suggesting that the formation of atomic oxygen with the support of the catalysis of Pt-BE is not crucial. It is also noted that the result shown in Fig. 3(b) suggests that catalytic ability of cathodes is not necessary for the occurrence of reset.

**Figure 4:**
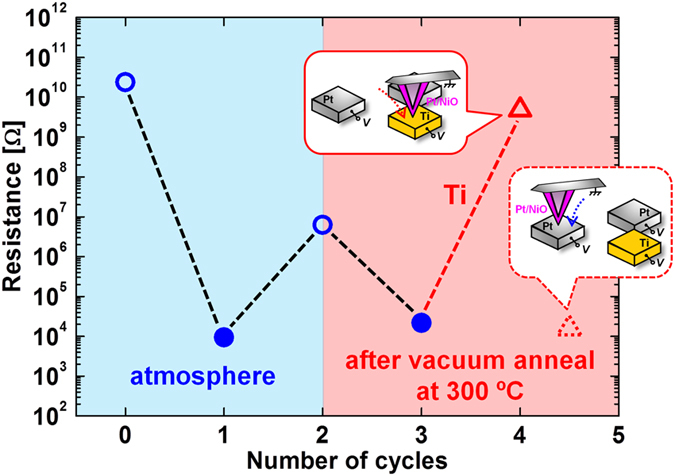
Sequential resistive switching: forming on Pt-BE(FS) ⇒ reset on Pt-BE(R) ⇒ vacuum anneal for 10 min at 300 °C ⇒ set on Pt-BE(FS) ⇒ reset on Ti-BE(R) ⇒ set on Pt-BE(FS), where all the measurements after the vacuum annealing were performed at RT without breaking the vacuum. Resistance values after set and reset are shown respectively by filled and open symbols. After the occurrence of reset on the Ti-BE(R) (open triangle), resistance value was read out again on the Pt-BE(FS), and the readout value is shown by dotted-line triangle.

On the other hand, significant results were observed when a Ti-BE(R) was used. We performed the same experiment by using a substrate on which a Ti-BE(R) was formed with Pt-BE(FS) and Pt-BE(R), which is shown in Fig. 1(c). The ΔG of Ti is shown in Table 1. After confirming the occurrence of forming and the first reset on the Pt-BEs in the atmosphere as shown respectively by the first filled circle and second open circle, both the Pt- and Ti-BEs were annealed in vacuum for 10 min at 300 °C and cooled to RT. The occurrence of the first set was observed as shown by filled circle in Fig. 4. Next, the cantilever was moved to the Ti-BE, and reset was attempted. The occurrence of reset was confirmed on the Ti-BE(R) at an average pulse height of 0.80 V and, therefore, the readout value of the resistance was high enough to be recognized as a high resistance state (HRS) as shown by the open triangle in Fig. 4. However, this high resistance value was not maintained when the cantilever was moved to the Pt-BE(FS) without applying a voltage. The readout value of the resistance on the Pt-BE(FS) was low enough to be judged as a low resistance state (LRS), as shown by the dotted-line triangle in Fig. 4.

This series of results suggest that when an anode with a high ΔG is used, reset occurs owing to the migration of oxygen ions inside the NiO layer, whereas when an anode with a low ΔG is used, reset occurs owing to oxidation of the anode. Therefore, the NiO layer itself works as an OR for anodes with a high ΔG, whereas the anode itself works as an OR for anodes with a low ΔG. It is widely accepted that CFs consist of V_O_’s, and reset is caused by V_O_ migration due to the V_O_ concentration gradient. On the basis of this reset mechanism, set could occur owing to the electric field drift of V_O_’s when an anode with a low ΔG is used. In this case, a reset and set are caused by the exchanging of V_O_’s between the CF and the anode in a direction perpendicular to the electrode interface^12^. However, the anode with a ΔG that is much lower than the ΔG of Ni in anode/NiO/cathode structures deprives oxygen from the NiO layer. As a result, highly insulating oxide is formed at the surface of the anode and prevents the occurrence of resistive switching^19^. On the other hand, because the OR was suggested to be an NiO layer itself when an anode with a high ΔG is used as an anode, set and reset are to be repeated by exchanging V_O_’s between a CF and an NiO layer excluding the CF. Therefore, a driving force that is different from an electric field drift and that works in a direction parallel to the electrode interface is required to cause set switching when an anode with a high ΔG is used. One of the candidates for this driving force is the Soret force, which works in the direction of the temperature gradient^20^,^21^,^22^,^23^,^24^. In this case, resistive switching takes place simply by the modification of the V_O_ distribution inside the NiO layer and, therefore, good switching endurance is ensured.

**Figure 5:**
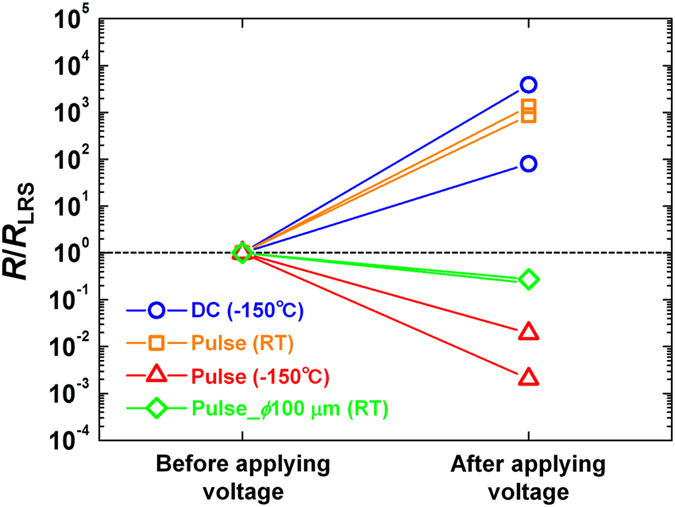
Resistance before and after the attempt to reset Pt cantilever/Pt-BE(R) structures by ramping voltage from 0 up to 3 V with sweep rate of ~5 V/s at −150°C (circles) and by applying voltage pulse with rise time of 2 μs, which corresponds to sweep rate of ~10^6^ V/s, at room temperature (squares) and −150°C (triangles). Diamonds show resistance before and after the attempt to reset normal Pt/NiO/Pt stack structures with the area of 100 μm in diameter by applying a voltage pulse with the rise time of 2 μs at room temperature.

Figure 5 shows resistance before and after the attempt to reset Pt cantilever/Pt-BE(R) structures by ramping the voltage up from 0 to 3 V with a sweep rate of ~5 V/s and by applying a voltage pulse with a rise time of 2 μs, which corresponds to a sweep rate of ~10^6^ V/s. The occurrence of reset was confirmed both after applying the sweep voltage at −150 °C (circle in Fig. 5) and after applying the voltage pulse at RT (square in Fig. 5) as well as after applying the sweep voltage at RT as shown in Fig. 2. On the other hand, reset did not occur after applying the voltage pulse at −150 °C (triangles in Fig. 5). The resistance became rather low compared with the resistance before applying the voltage pulse. This result can be explained by assuming that the driving force causing set switching is the Soret force. The fluxes of oxygen vacancies for Fick diffusion *J*_Fick_ and for Sorret diffusion *J*_Soret_ can be expressed as


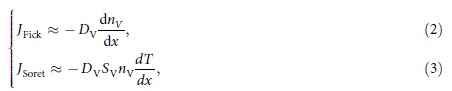


where *D*_V_, *n*_V_, and *S*_V_ are the diffusion constant, the density, and the Soret coefficient of the vacancies as a function of the radial coordinate, *x*, respectively, if a cylindrical cell structure is assumed. Here, the Soret coefficient is defined as the ratio of the thermal diffusion constant and the normal diffusion constant^25^ and must be negative for V_O_’s^24^. Assuming that the CF consists of V_O_’s^26^,^27^, they diffuse from the CF to the outside of the CF by Fick diffusion because V_O_’s diffuse from high- to low-V_O_ concentration areas according to Equation 2, generating *J*_Fick_ and causing reset switching. On the other hand, V_O_’s diffuse toward a current path that is a heating center owing to Joule heating from the surrounding area by Soret diffusion because V_O_’s diffuse from low- to high-temperature areas according to Equation 3, generating *J*_Soret_ and causing set switching. So, *J*_Fick_ and *J*_Soret_ are always competing during resistive switching. When the voltage sweep rate is small enough to maintain thermal equilibrium continuously, *J*_Soret_ is small because the temperature gradient, d*T*/dx, in Equation 3 is small. However, when the voltage sweep rate becomes large so that thermal equilibrium cannot be maintained, the larger the voltage sweep rate becomes, the larger d*T*/d*x* becomes. *J*_Soret_, therefore, becomes dominant for larger voltage sweep rates. In addition, because *S*_Vo_ is expressed as -*U*/(*k*_B_*T*^2^), the ratio of *J*_Fick_ to *J*_Soret_ is proportional to *T*^2^, i.e., |*J*_Fick_/*J*_Soret_| ∝ *T*^2^, where *U* is the energy barrier between the potential wells for V_O_ diffusion, and *k*_B_ is the Boltzmann constant^24^. This means that *J*_Soret_ becomes dominant at low temperatures and hinders reset switching. Therefore, it is expected that it will be difficult for reset to occur when a voltage pulse with a short rise time is applied at a low temperature, which is consistent with the result shown in Fig. 5.

**Figure 6:**
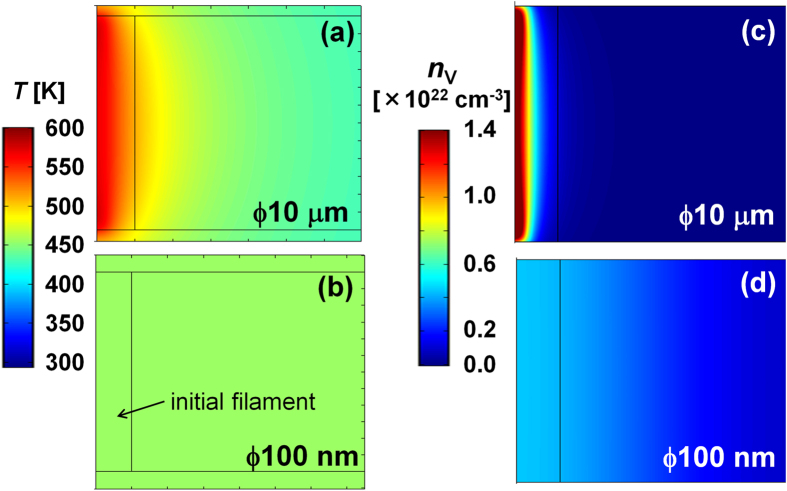
Simulated temperature distribution in Pt/NiO/Pt stack structures with (a) large (10 μm in diameter) and (b) small (100 nm in diameter) area, respectively, at 1.01 V during application of a voltage pulse with the rise time of 2 μs and the pulse height of 1.20 V. A filament consisting of V_O_’s with the radius of 10 nm is located at the center before the voltage application in both large and small structures. *n*_V_ distribution in (**c**) large and (**d**) small structures for the temperature distributions (**a**) and (**b**), respectively.

On the other hand, we also measured resistance before and after attempt to reset normal Pt/NiO/Pt stack structures with the area of 100 μm in diameter by applying a voltage pulse with the rise time of 2 μs and the pulse height of 1.2 V at RT (diamonds in Fig. 5). In this case, reset did not occur and the resistance rather decreased in contrast to the case using Pt cantilever/Pt-BE(R) structures at RT. Figure 6(a,b) show simulated temperature distribution in Pt/NiO/Pt stack structures with large (10 μm in diameter) and small (100 nm in diameter) area, respectively, at 1.01 V during application of a voltage pulse with the rise time of 2 μs and the pulse height of 1.20 V (Fig. S2). A CF consisting of V_O_’s with the radius of 10 nm is located at the center before the voltage application (for details, see supplemental information). Temperature gradient near the CF is steep in the large structure, whereas relatively flat in the small structure due to the accumulation of Joule heat. As a result, in the large structure, oxygen vacancies diffuse toward the center of the CF by Soret diffusion as shown in Fig. 6(c), causing set switching. On the other hand, in the small structure, oxygen vacancies diffuse outward from the CF by Fick diffusion, and oxygen vacancy concentration is flattened as shown in Fig. 6(d), causing reset switching. Therefore, the simulation result is consistent with the experimentally obtained data above, supporting that Soret diffusion is the driving force of set switching.

In conclusion, the present work provided a picture of the occurrence of resistive switching: the location of the OR differs depending on the ΔG of the anode material, and resistive switching occurs by the exchange of V_O_’s between a CF and the OR. An OR is a NiO layer excluding the CF for high ΔG values, whereas it is an anode for low ΔG values. This clearly suggests that the location of the OR depends on the relative magnitude of the Gibbs energies of the anode material and the metal M of an MO layer. The presence of a driving force in a direction parallel to the electrode interface is required to cause set switching when an anode with a high ΔG is used. Elucidating the driving force is crucial for a deeper understanding of the resistive switching mechanism.

## Methods

### Sample preparation

A Pt or Au film with a thickness of 20 nm was deposited on an AFM cantilever with a tip radius of 50 nm (Hitachi High-Technologies, SI-DF3-R(100)) as the TE, followed by the deposition of an NiO film with a thickness of 15 nm as a memory layer at RT by using the DC reactive magnetron sputtering method in a mixture of Ar + O_2_ gases. During the NiO deposition, the pressure of the mixture gas was maintained at 0.50 Pa (Ar:O_2_ = 0.42:0.08 Pa). The cantilever on which a Pt/NiO or an Au/NiO structure was formed was described respectively as a Pt cantilever or an Au cantilever in this paper. We also prepared normal Pt(100 nm)/NiO(60 nm)/Pt(100 nm) stack structures. 100 nm thick Pt-TEs with the area of 100 μm in diameter were deposited by DC sputtering using a shadow mask, after NiO deposition by DC reactive sputtering method at 380 °C in the mixed gas of Ar and O_2_ gasses (Ar : O_2_ = 0.45 Pa : 0.05 Pa).

Pt-BE(FS) and Pt-, Au-, Ni-, and TiN-BE(R)s with a thickness of 100 nm were prepared on the same SiO_2_(100 nm)/Si(650μm) substrate by a sputtering method (Fig. 1(a)). In addition, Pt-BE(FS) and Ti- and Pt-BE(R)s with a thickness of 100 nm were prepared on the another same SiO_2_(100 nm)/Si(650 μm) substrate by a sputtering method (Fig. 1(c)). This substrate was annealed under a reductive atmosphere of H_2_ and Ar mixture gas (3.0% of H_2_) for 5 min at 300°C for the reduction of the native oxide film that was formed on the surface of Ti-BE(R).

### Electric characteristics measurements

A bias voltage was applied between the cantilever and the BE by using a pulse generator (Agilent 81110A) or a source measure unit (Keythley 236) in the contact mode of the AFM. A reset was attempted by applying pulse voltages with the duration of 100 μs. The pulse height was increased from 0.8 V in steps of 0.1 V until a voltage was reached at which the occurrence of reset was confirmed. On the other hand, forming and set were performed by sweeping the voltage to 10 V and to 8 V, respectively. By sequentially contacting each BE with the same cantilever, the effect of replacing anodes on the resistive switching property was investigated. The bias voltage was applied to the BEs, whereas a cantilever that works as a TE was grounded. The bias voltage was always positive in this study: we attempted only unipolar switching. The AFM system used in this study (Hitachi High-Technologies, E-sweep) was equipped with a vacuum chamber and a heater in the chamber so that the atmospheric pressure and temperature were controllable. Forming and the first reset were performed on the Pt-BE(FS) and the Pt-BE(R) in the atmosphere, respectively. After that, all switching was performed in vacuum (~10^−4^ Pa). A substrate temperature was controlled within the range −150 °C to 300 °C.

### Simulation

Reset switching was attempted by simulating V_O_ migration using commercial software (COMSOL Multiphysics). We assumed Soret and Fick diffusion, whereas electric field drift and Fick diffusion are generally adopted as driving forces of V_O_ migration. For the details about simulation, see supplemental information.

## Additional Information

**How to cite this article**: Kinoshita, K. *et al.* Finding Oxygen Reservoir by Using Extremely Small Test Cell Structure for Resistive Random Access Memory with Replaceable Bottom Electrode. *Sci. Rep.* **5**, 18442; doi: 10.1038/srep18442 (2015).

## Supplementary Material

Supplementary Information

## Figures and Tables

**Table 1:**
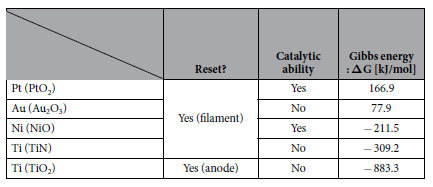
The presence of catalytic ability and Gibbs free energy of formation reaction (ΔG) at room temperature of the bottom electrodes (BEs) used in this study, where ΔG is defined as the difference between the Gibbs free energy of metal oxide or metal nitride indicated in the parenthesis and the Gibbs free energy of pure metal (Pt, Au, Ni, and Ti).
