# Supplementary material for: Finding Oxygen Reservoir by Using Extremely Small Test Cell Structure for Resistive Random Access Memory with Replaceable Bottom Electrode
Source: Sci Rep. 2015 Dec 22;5:18442. doi: 10.1038/srep18442 (PMC4686884; doi:10.1038/srep18442)
Supplement: Supplementary Information [file srep18442-s1.pdf]

Supplementary Information

Finding Oxygen Reservoir by Using Extremely Small Test Cell Structure for Resistive Random Access Memory with Replaceable Bottom Electrode

Kentaro Kinoshita[1,2,3, *], Sang-Gyu Koh[1], Takumi Moriyama[1,2], and Satoru Kishida[1,2,3]

[1]Department of Information and Electronics, Graduate School of Engineering, Tottori University, 4-101 Koyama-Minami, Tottori 680-8552, Japan.

[2]Tottori Integrated Frontier Research Center, 4-101 Koyama-Minami, Tottori 680-8552, Japan.

[3]Tottori Univ. Electronic Display Research Center, 4-101 Koyama-Minami, Tottori 680-8552, Japan.

*E-mail: kinoshita@ele.tottori-u.ac.jp

Simulation

Simulation conditions.

Simulation was performed using commercial software COMSOL Multiphysics. We used cylindrical Pt/NiO/Pt cells with the area of 10 μm (large cell) and 100 nm (small cell) in diameter, as shown in Fig. S1, where the dashed line was symmetry axis. A filament (FL) consisting of oxygen vacancies, V_O's, with the radius of 10 nm is located at the center. The memory cell was surrounded by 10 μm thick air and temperature is fixed at 293 K at the edge of the air region. Vo concentration, n_V, at the filament and out of the filament are shown in Table S1. We attempted to reset the large and small cells by applying a pulse voltage with the rising time and the pulse height of 1.8 μs and 1.2 V, respectively (Fig. S2).

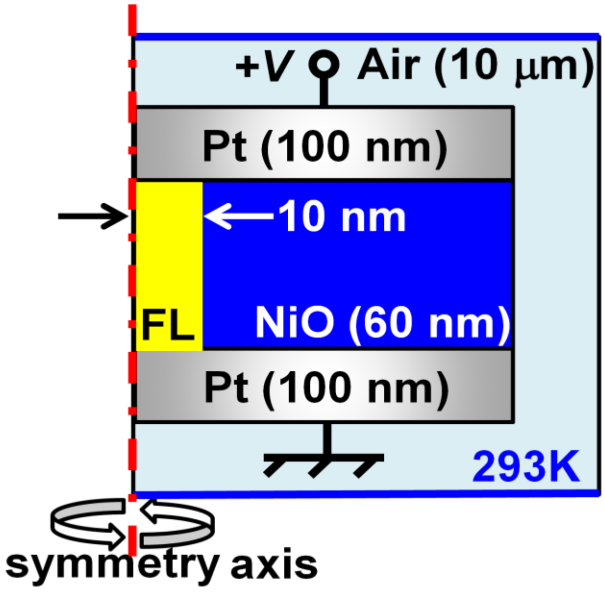

Figure S1. Simulation model used in this study.

Calculation flow (all equations are summarized in Equations section below).

(1) The initial value of n_V (Table 1) and time dependence of applied voltage, $V(t)$, (Fig. S2) were given.

(2) Electric conductivity, σ, is calculated by Equation (1) using the given n_V by assuming pre-factor σ_0 is proportional to n_V (Fig. S3).

(3) Electric field, $\boldsymbol{E}$, and electric current density, $\boldsymbol{j}$, is calculated by solving Equations (2)-(4), where Equation (4) is a current continuity equation for electrical conduction.

(4) T distribution is calculated by solving heat equation (Equation (5)), where Equation (6) was used as temperature dependence of C_p and the inner product of $\boldsymbol{j}$ and $\boldsymbol{E}$ gives Joule heat.

(5) Flux of Fick diffusion, $\boldsymbol{J}_{fick}$, and Soret diffusion, $\boldsymbol{J}_{soret}$, are calculated respectively by solving Equations (7) and (8) using n_V and T distributions. D_V is given by Equation (9).

(6) Updated n_V after dt sec was calculated by solving V_O transport equation (Equation (10)).

(7) (2)-(6) are repeated.

Equations

(1) Electric conductivity: $\sigma = \sigma_0 \cdot exp\left(-\frac{E_a}{k_B T}\right)$. (1)

(2) Electric field: $\boldsymbol{E} = \nabla V$. (2)

(3) Electric current density: $\boldsymbol{j} = \sigma \boldsymbol{E}$. (3)

(4) Current continuity equation for electrical conduction: $\nabla \cdot \boldsymbol{j} = 0$. (4)

(5) Heat equation: $\boldsymbol{\rho C_\mathrm{p}} \frac{\boldsymbol{\partial n_\mathrm{V}}}{\boldsymbol{\partial t}} - \boldsymbol{\nabla} \cdot (\boldsymbol{k} \nabla \boldsymbol{T}) = \boldsymbol{j} \cdot \boldsymbol{E}$. (5)

(6) Isobaric specific heat capacity of NiO: $C_\mathrm{p} = 10.563 \cdot \exp\left(\frac{T}{140.3}\right) + 506.64$. (6)

(7) Flux of Fick diffusion: $\boldsymbol{J}_\mathbf{Fick} = -\boldsymbol{D}_\mathbf{V} \nabla \boldsymbol{n}_\mathbf{V}$. (7)

(8) Flux of Soret diffusion: $\boldsymbol{J}_\mathbf{Soret} = -\boldsymbol{D}_\mathbf{V} \frac{\boldsymbol{U}_\mathbf{a}}{\boldsymbol{kT^2}} \boldsymbol{n}_\mathbf{V} \nabla \boldsymbol{T}$. (8)

(9) Diffusion coefficient for V_O transport: $D_\mathrm{V} = \frac{1}{2} a^2 f \exp\left(-\frac{U_\mathrm{a}}{\kappa_\mathrm{B} T}\right)$. (9)

(10) Diffusion equation for V_O transport: $\frac{\boldsymbol{\partial n_\mathrm{V}}}{\boldsymbol{\partial t}} = \nabla \cdot (\boldsymbol{J}_\mathbf{Fick} + \boldsymbol{J}_\mathbf{Soret})$. (10)

All numeric values for parameters used in the simulation were summarized in Table S1.

Constants

M molar mass of NiO
⇒ 74.7 g/mol [1)]

n_V concentration of Vo (filament region)
⇒ 5.38×10^{22} cm^{-3} (max.), 10 cm^{-3} (min.), 6.86×10^{21} Ω^{-1}m^{-1} (ini.: 200Ω)

E_a activation energy for electric conduction
⇒ 0.381 eV (n_V = 10 cm^{-3}), 0 eV ($n_V \geq 2.69\times10^{21}$ cm^{-3})

U_a activation energy for V_O diffusion
⇒ 0.975 eV [2)]

σ_0 coefficient of conductivity of NiO
⇒ 1.44×10^{7} Ω^{-1}m^{-1} ($n_V = 5.38 \times 10^{22}$ cm^{-3}) [1)], 1.90×10^{-3} Ω^{-1}m^{-1} (n_V = 10 cm^{-3})

k thermal conductivity
⇒ 90.5 Wm^{-1}K^{-1} (Ni) [1)], 71.0 Wm^{-1}K^{-1} (NiO) [1)], 70.0 Wm^{-1}K^{-1} (Pt) [1)]

C_p thermal capacitance of NiO
⇒ 595.4 Jkg^{-1}K^{-1} (300℃) [3)], 689.5 Jkg^{-1}K^{-1} (400℃) [3)],
869.9 Jkg^{-1}K^{-1} (500℃) [3)], 947.1 Jkg^{-1}K^{-1} (525℃) [3)]

C_p thermal capacitance of Pt
⇒ 134.0 Jkg^{-1}K^{-1} [4)]

ρ mass density
⇒ 6670 kg/m^3 (NiO) [1)], 2145×10^4 kg/m^3 (Pt) [5)]

α temperature coefficient of Pt
⇒ 3.9×10^{-3} K^{-1} [1)]

ρ_0 resistivity of Pt
⇒ 10.6×10^{-8} Ωm (293.15 K) [1)]

Table S1. Numeric values for parameters used in this study.

Parameters from measurements and assumptions.

To solve the heat equation (Equation (5)), electric conductivity, σ, thermal conductivity, k, thermal capacitance C_p, and mass density ρ of the NiO memory layer including V_O filament and Pt electrodes have to be decided. Furthermore, n_V also should be decided to solve equations for fluxes of Fick and Soret diffusion of V_O (Equations (7) and (8)).

Parameters of memory layer (NiO and V_O filament).

V_O concentration n_V.

A filament in the NiO layer of a Pt/NiO/Pt structure is generally considered to be formed by V_O's. This means that a filament is area where V_O concentration, n_V, is higher than surrounding area and the n_V. We assumed that n_V can change from the value of NiO before forming (minimum n_V) continuously to the value of completely V_O-depleted NiO (maximum n_V). The maximum and minimum n_V values were assumed to be 5.38×10^{22} cm^{-3} and 10 cm^{-3}, respectively. The former and the latter correspond to n_V values of completely V_O-depleted NiO crystal and almost perfect NiO crystal, respectively.

Electric conductivity.

Electric conductivity of NiO is given by the Arrhenius equation (Equation (1)), where pre-factor σ_0 and activation energy for electric conduction E_a. We assumed the n_V dependences of σ_0 and E_a as shown in Figs. S3 (a) and (b), respectively. σ_0 increases linearly with increasing n_V from σ_0 = 1.9×10^{-3} Ω^{-1}m^{-1} at the minimum nv to σ_0 = 1.44×10^{7} Ω^{-1}m^{-1} at the maximum n_V. On the other hand, E_a decreases linearly with increasing nv from 0.381 eV at the minimum nv to 0 eV at nv = 2.69×10^{21} cm^{-3} (1/20 of maximum nv), and E_a = 0 eV for nv $\geq 2.69 \times 10^{21}$ cm^{-3}. Here, E_a at the minimum nv value of 0.381 eV was estimated from the Arrhenius plot of the experimentally obtained temperature dependence of ρ in Fig. S4(b) that was estimated by the relation of $\rho = R_{ini}\times S/d$ using the temperature dependence of resistance of a Pt/NiO/Pt structure in the initial state, R_{ini}, the cell area, S, of ϕ150 μm in diameter, and the NiO thickness, d, of 60 nm. The absolute value of σ_0 at the minimum nv was decided to be 1.9×10^{-3} Ω^{-1}m^{-1} by Equation (1) using σ (= $1/\rho$) at 298 K, 14.7 μΩ^{-1}m^{-1}, and E_a of 0.381 eV estimated above.

Thermal conductivity

Thermal conductivity, k, was assumed to be linearly dependent on n_V as shown in Fig. S5. k increases from 71 $Wm^{-1}K^{-1}$ [6], which is k of NiO, for the minimum n_V linearly to 90.5 $Wm^{-1}K^{-1}$ [1], which is k of Ni, for the maximum n_V.

Isobaric specific heat capacity

Temperature dependence of isobaric specific heat capacity was estimated by interpolation and extrapolation of experimentally obtained C_p values of NiO[3] shown by circles in Fig. S6. The data was fit well to Equation (6). Temperature dependence of isobaric specific heat capacity was estimated by interpolation and extrapolation using Equation (6).

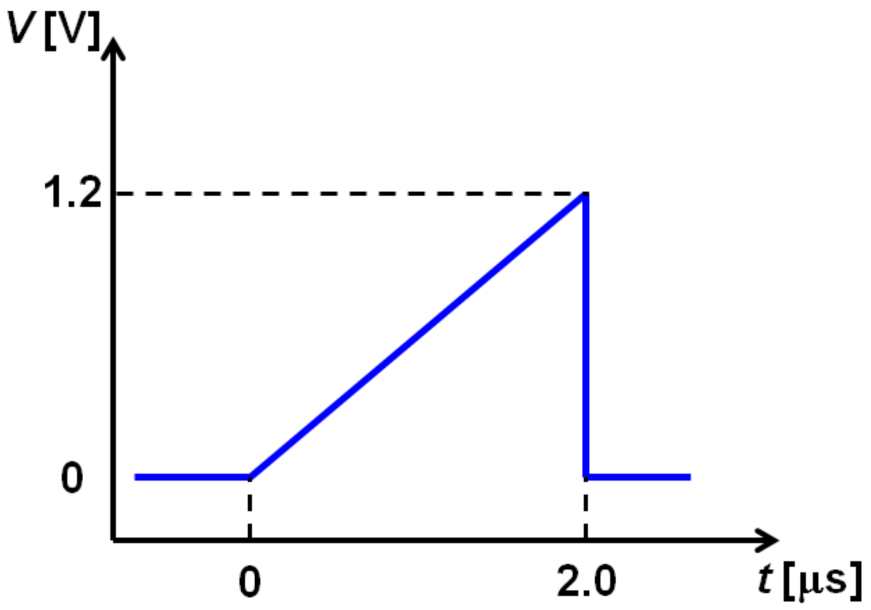

Figure S2. Time dependence of applied voltage, $V(t)$. Rising time, t_{rise}, and pulse height, V_p, were 2.0 μs and 1.2 V, respectively.

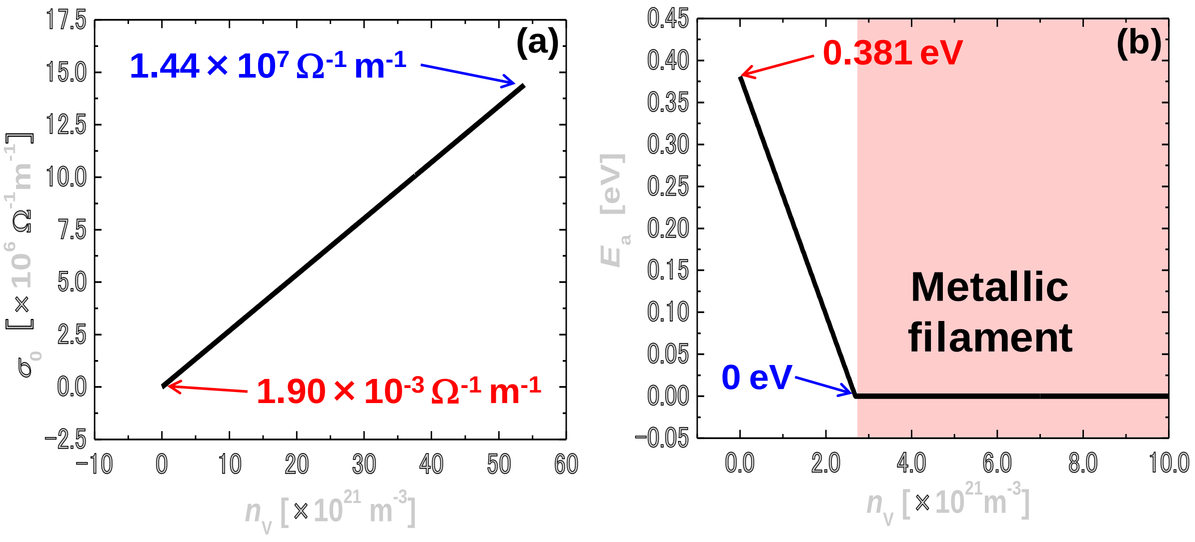

Figure S3. Assumed (a) n_V dependences of electrical conductivity pre-exponential factor, σ_0, and (b) activation energy for conduction, E_a.

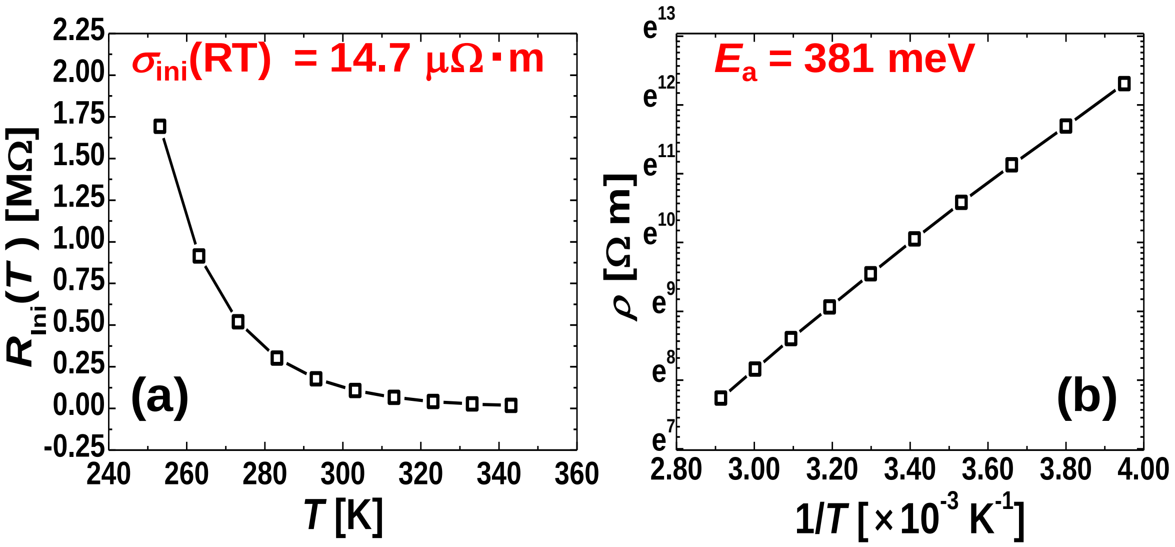

Figure S4. (a) Measured temperature dependence of resistance of a Pt/NiO/Pt structure in the initial state, R_{ini}, the cell area, S, of ϕ150 μm in diameter, and the NiO thickness, d, of 60 nm. (b) The Arrhenius plot of the temperature dependence of ρ that was estimated by the relation of $\rho = R_{ini} \times S / d$ using $R_{ini}(T)$ in Fig. S4(a), the cell area, S, of ϕ150 μm in diameter, and the NiO thickness, d, of 60 nm.

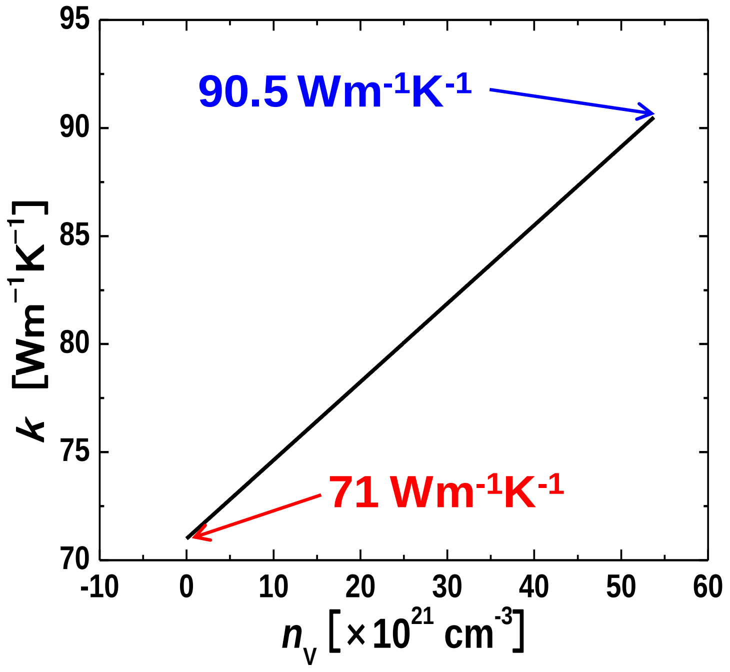

Figure S5. Assumed n_V dependence of thermal conductivity.

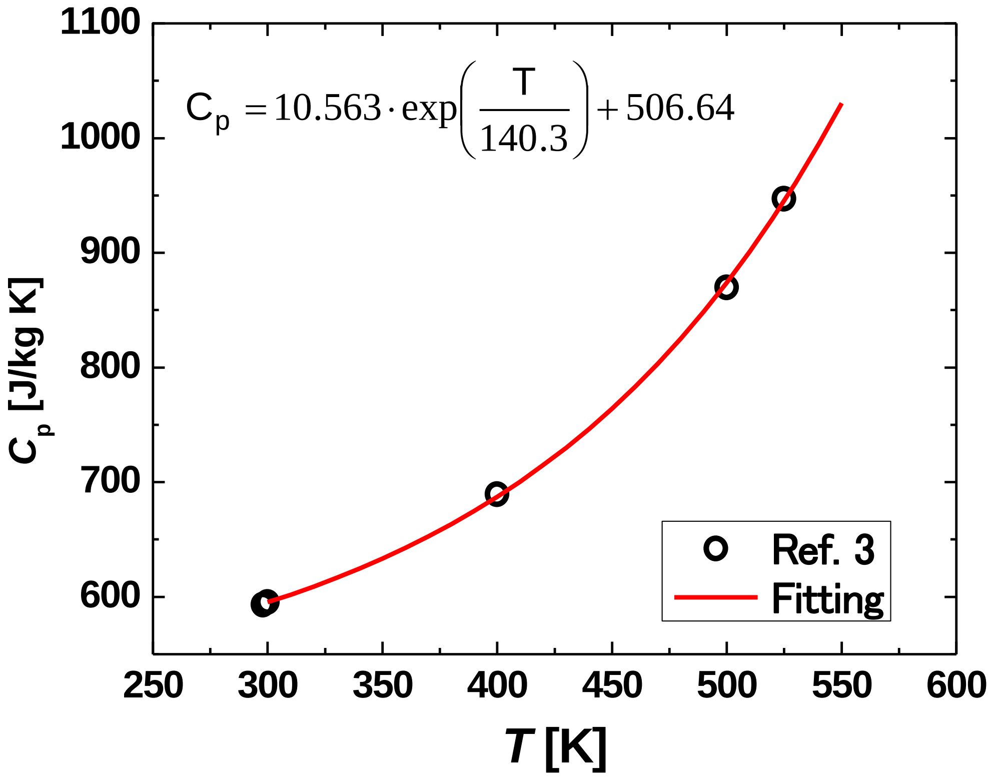

Figure S6. Experimental (circles) [3)] and fitting results (line).

References

1. Chronological scientific tables (Maruzen, 2014).

2. Nowotny, J. and Sadowski, A. Chemical Diffusion in Nickel Oxide. J. Am. Ceram. Soc. **62**, 1 (1979).

3. Barin, I. Thermochemical Data of Pure Substances 98-1547 (VCH, 1989).

4. Furukawa, G.T. *et al*. Critical Analysis of Heat-Capacity Data and Evaluation of Thermodynamic Properties of Ruthenium, Rhodium, Palladium, Iridium, and Platinum from

0 to 300 K. A Survey of the Literature Data on Osmium. J. Phys. Chem. Ref. Data **3**, 1 (1974).

5. Ishikawa, T. *et al*. Jpn. J. Appl. Phys. 45, 1719 (2006).

6. Sato, Y. *et al*. Consideration of switching mechanism of binary metal oxide resistive junctions using a thermal reaction model. Appl. Phys. Lett. **90**, 033503 (2007).
